# Supplementary material for: Localization of potato browning resistance genes based on BSA-seq technology
Source: PeerJ. 2024 Aug 6;12:e17831. doi: 10.7717/peerj.17831 (PMC11313402; doi:10.7717/peerj.17831)
Supplement: Table S1 [file peerj-12-17831-s003.docx]

Table S1 275 Potato Resources

| Number | Name | Number | Name | Number | Name |
| --- | --- | --- | --- | --- | --- |
| 1 | -9 | 93 | Hu H97-9 | 185 | Pepo416 |
| 2 | 194 | 94 | Hu H99-1 | 186 | Pepo418 |
| 3 | 295 | 95 | Hu H99-9 | 187 | Pepo426 |
| 4 | 04P48-3 | 96 | Hu83210 | 188 | Qibaiwan |
| 5 | 05HE5-43 | 97 | Hu9058-2 | 189 | Qingshu 9 |
| 6 | 06H26 | 98 | IH01-5 | 190 | Quarta |
| 7 | 08CA0979 | 99 | IMRALA-1 | 191 | s.goniocalyx |
| 8 | 08CA9728-04 | 100 | J10828 | 192 | S_5-2-7-44-1-10-5-2-1-1-6-1-(11)_ |
| 9 | 08HE042-2 | 101 | Jin90-7-23 | 193 | Saikai35 |
| 10 | 2013Y45 | 102 | Jizhang | 194 | Sante |
| 11 | 292-20 | 103 | Jizhang 11 | 195 | sebago |
| 12 | 83Y64 | 104 | Jizhang 14 | 196 | SH11R-6 |
| 13 | Amsel | 105 | Jizhang 8 | 197 | Shancheng |
| 14 | Andover | 106 | Jizhang12 | 198 | shepody |
| 15 | Anemone | 107 | K1 | 199 | Solist |
| 16 | Aquila | 108 | K16-6 | 200 | spunta |
| 17 | Astilla | 109 | K2 | 201 | Superior |
| 18 | Atlantic | 110 | K200001-24 | 202 | Taihe |
| 19 | Atzimba | 111 | K200373-13 | 203 | Vester |
| 20 | Aula | 112 | K200632-12 | 204 | Villas |
| 21 | Ba 90-2-6 | 113 | K200809-90 | 205 | Vitara |
| 22 | Bashu 7 | 114 | K200846-10 | 206 | Weishu 1 |
| 23 | BE200158-3 | 115 | K200852-13 | 207 | WYJ1 |
| 24 | BE200170-10 | 116 | K200852-205 | 208 | WYJ2 |
| 25 | BE20079-24 | 117 | K200852-24 | 209 | WYJ3 |
| 26 | BE20413-22 | 118 | K200856-6 | 210 | WYJ4 |
| 27 | Bintje | 119 | K200858-22 | 211 | WYJ5 |
| 28 | Bo-B2 | 120 | K200867-15 | 212 | WYJ7 |
| 29 | Bo-BR | 121 | K200904-37 | 213 | WYJ8 |
| 30 | Bo-C | 122 | K200908-16 | 214 | WYJ9 |
| 31 | Bo-S | 123 | K200920-11 | 215 | WYJ11 |
| 32 | Boyou 2 | 124 | K200920-28 | 216 | WYJ12 |
| 33 | Cal White | 125 | K200920-35 | 217 | WYJ13 |
| 34 | Carola | 126 | K200927-13 | 218 | WYJ14 |
| 35 | Chenggong | 127 | K200931-1 | 219 | WYJ15 |
| 36 | Chuanyi | 128 | K200935-43 | 220 | Xindaping |
| 37 | Chunshu 1 | 129 | K200935-51 | 221 | Xingjia 2 |
| 38 | CIP388611.22 | 130 | K200938-48 | 222 | Xisen 3 |
| 39 | CIP388615.22 | 131 | K200950-3 | 223 | Xisen 4 |
| 40 | CIP388676.1 | 132 | K200961-5 | 224 | Yan 0225-432 |
| 41 | CIP388972.22 | 133 | K200968-12 | 225 | Yan 2005-1 |
| 42 | CIP390663.8 | 134 | k200969-11 | 226 | Yanshu 3 |
| 43 | CIP391002.6 | 135 | K200969-2 | 227 | Yanshu 4 |
| 44 | CIP391011.17 | 136 | K200969-4 | 228 | Yiselie |
| 45 | CIP391180.6 | 137 | K200979-17 | 229 | Youjin |
| 46 | CIP392633.54 | 138 | K200979-25 | 230 | Yunshu 103 |
| 47 | CIP392797.22 | 139 | K200979-3 | 231 | Yunshu 201 |
| 48 | CIP393077.159 | 140 | K3 | 232 | Yunshu 202 |
| 49 | CIP393077.54 | 141 | K4 | 233 | Yunshu 205 |
| 50 | CIP393371.157 | 142 | K9201-10 | 234 | Yunshu 303 |
| 51 | CIP393615.6 | 143 | K9412-13 | 235 | Yunshu 401 |
| 52 | CIP393617.1 | 144 | Kangheijingbing | 236 | Yunshu 501 |
| 53 | CIP395037.107 | 145 | Kangyibai | 237 | Yunshu 505 |
| 54 | CIP395109.29 | 146 | katadin | 238 | Yunshu827 |
| 55 | CIP395112.9 | 147 | Kende | 239 | Zaodabai |
| 56 | CIP395434.1 | 148 | Kennebec | 240 | Zhengshu 5 |
| 57 | CIP396004.263 | 149 | Kexin 1 | 241 | Zhong A9215-84 |
| 58 | CIP396033.102 | 150 | Kexin 2 | 242 | Zhong C9305-6 |
| 59 | CIP396311.1 | 151 | Kexin 4 | 243 | Zhongshu 1 |
| 60 | CIP397073.16 | 152 | Kexin 6 | 244 | Zhongshu 4 |
| 61 | CIP397077.16 | 153 | Kexin 12 | 245 | Zhongshu 5 |
| 62 | CIP397100.9 | 154 | Kexin 13 | 246 | Zhongshu 6 |
| 63 | CIP399004.19 | 155 | Kexin 14 | 247 | Zhongshu 12 |
| 64 | CIP703831 | 156 | Kexin 17 | 248 | Zhongshu 17 |
| 65 | Ckapб | 157 | Kexin 18 | 249 | Zhukefu |
| 66 | Colmo | 158 | Kexin 19 | 250 | Архидея |
| 67 | Dadi | 159 | Kexin 20 | 251 | Атлант |
| 68 | Delta | 160 | Kexin 21 | 252 | Вилъня |
| 69 | Denali | 161 | Kexin 22 | 253 | вир244(Suniax-S.Stolonig) |
| 70 | Dianella | 162 | Kexin 23 | 254 | вишневый |
| 71 | Diniela | 163 | Kexin 25 | 255 | выток |
| 72 | Dongnong 00-33048 | 164 | Kexin 26 | 256 | гибрид 59/m-56 |
| 73 | Dongnong 0733-125 | 165 | Khongor | 257 | гибрид 728-6 |
| 74 | DY4-5-10 | 166 | Kunta | 258 | гибрид 90.1/11 |
| 75 | E-13 | 167 | KURODA | 259 | гибрид МВ-168 |
| 76 | E-3 | 168 | Long201207-4 | 260 | гибрид59/m-69 |
| 77 | E-421 | 169 | Long201208-13 | 261 | гибрид80-1 |
| 78 | E-5 | 170 | Longshu 3 | 262 | гибридMB-168 |
| 79 | Eramosa | 171 | Longshu 6 | 263 | Дельфин |
| 80 | Eyin | 172 | Longshu 7 | 264 | Журавинка |
| 81 | F00070 | 173 | Longxinxuan 1 | 265 | зарево |
| 82 | F70021-1 | 174 | Lt-5 | 266 | Зда**б**ыток |
| 83 | Favorita | 175 | Maiken 1 | 267 | Лилея |
| 84 | Feixing | 176 | Maiken 4 | 268 | Маг |
| 85 | FL1533 | 177 | Maiken 5 | 269 | Максимум |
| 86 | FL1625 | 178 | Minshu 1 | 270 | Московский |
| 87 | Fujian | 179 | Nanjue | 271 | никунский |
| 88 | GADRE | 180 | Ne 16 | 272 | орбита |
| 89 | GaRant(mapaиm) | 181 | Nehe gaodianfen | 273 | Ранный |
| 90 | Hu 5 | 182 | Norland | 274 | Тарант |
| 91 | Hu 8212-3 | 183 | NS51-5 | 275 | Уладар |
| 92 | Hu 9707-116 | 184 | NS78-11 |  |  |
