# Supplementary material for: Localization of potato browning resistance genes based on BSA-seq technology
Source: PeerJ. 2024 Aug 6;12:e17831. doi: 10.7717/peerj.17831 (PMC11313402; doi:10.7717/peerj.17831)
Supplement: Table S2 [file peerj-12-17831-s004.docx]

Table S2 Browning area of Level

| level | Browning area  (%) |
| --- | --- |
| 0 | area <25 |
| 1 | 25≤area <50 |
| 3 | 50≤area <75 |
| 4 | area≥75 |
