# Supplementary material for: Localization of potato browning resistance genes based on BSA-seq technology
Source: PeerJ. 2024 Aug 6;12:e17831. doi: 10.7717/peerj.17831 (PMC11313402; doi:10.7717/peerj.17831)
Supplement: Table S3 [file peerj-12-17831-s005.docx]

Table S3 Construction of potato populations with light and dark browning

| Number | Browning light（Q） | | | | | | | Number | Browning dark（Z） | | | | | | |
| --- | --- | --- | --- | --- | --- | --- | --- | --- | --- | --- | --- | --- | --- | --- | --- |
|  | 2021 | | |  | 2022 | | |  | 2021 | | |  | 2022 | | |
|  | Browning Index % | Browning Intensity | Cooking browning Index |  | Browning Index % | Browning Intensity | Cooking browning Index |  | Browning Index % | Browning Intensity | Cooking browning Index |  | Browning Index % | Browning Intensity | Cooking browning Index |
| T37 | 16.67 | 0.045 | 8.00 |  | 8.33 | 0.080 | 7.00 | T5 | 100.00 | 0.334 | 6.75 |  | 91.67 | 0.104 | 6.33 |
| T41 | 33.33 | 0.109 | 7.50 |  | 25.00 | 0.000 | 7.00 | T6 | 100.00 | 0.303 | 5.25 |  | 91.67 | 0.154 | 6.67 |
| T44 | 0.00 | 0.035 | 8.00 |  | 25.00 | 0.039 | 6.00 | T18 | 100.00 | 0.248 | 5.00 |  | 91.67 | 0.038 | 6.33 |
| T46 | 16.67 | 0.110 | 7.75 |  | 0.00 | 0.053 | 7.00 | T30 | 100.00 | 0.380 | 5.00 |  | 100.00 | 0.251 | 7.00 |
| T60 | 41.67 | 0.036 | 7.00 |  | 5.56 | 0.041 | 7.00 | T40 | 100.00 | 0.212 | 7.25 |  | 75.00 | 0.247 | 6.33 |
| T83 | 44.44 | 0.119 | 7.75 |  | 33.33 | 0.065 | 8.00 | T53 | 100.00 | 0.241 | 7.50 |  | 100.00 | 0.240 | 7.00 |
| T120 | 0.00 | 0.104 | 8.00 |  | 8.33 | 0.091 | 8.00 | T72 | 100.00 | 0.312 | 7.00 |  | 50.00 | 0.206 | 7.33 |
| T150 | 66.67 | 0.033 | 8.00 |  | 8.33 | 0.022 | 8.00 | T74 | 16.67 | 0.187 | 6.25 |  | 12.50 | 0.079 | 6.50 |
| T166 | 0.00 | 0.071 | 7.50 |  | 41.67 | 0.050 | 7.33 | T76 | 100.00 | 0.328 | 6.50 |  | 75.00 | -0.011 | 6.50 |
| T175 | 62.50 | 0.041 | 7.00 |  | 25.00 | 0.070 | 7.00 | T86 | 100.00 | 0.231 | 7.00 |  | 75.00 | 0.146 | 7.00 |
| T183 | 33.33 | 0.139 | 7.00 |  | 16.67 | 0.144 | 7.00 | T142 | 91.67 | 0.396 | 7.00 |  | 100.00 | 0.488 | 6.00 |
| T199 | 0.00 | 0.079 | 8.00 |  | 16.67 | 0.011 | 6.83 | T146 | 100.00 | 0.167 | 6.75 |  | 100.00 | 0.059 | 7.67 |
| T201 | 8.33 | 0.187 | 7.00 |  | 16.67 | 0.045 | 6.00 | T149 | 100.00 | 0.238 | 7.00 |  | 75.00 | 0.194 | 5.67 |
| T230 | 8.33 | 0.076 | 8.00 |  | 66.67 | 0.023 | 7.67 | T156 | 100.00 | 0.566 | 5.25 |  | 100.00 | 0.160 | 6.50 |
| T250 | 32.50 | 0.083 | 9.00 |  | 100.00 | 0.251 | 8.00 | T169 | 100.00 | 0.547 | 6.00 |  | 100.00 | 0.147 | 6.00 |
| T256 | 16.67 | 0.128 | 7.00 |  | 16.67 | 0.051 | 8.00 | T190 | 91.67 | 0.368 | 8.00 |  | 100.00 | 0.376 | 7.33 |
| T286 | 25.00 | 0.081 | 7.00 |  | 8.33 | 0.086 | 7.00 | T232 | 100.00 | 0.071 | 5.50 |  | 66.67 | 0.174 | 7.00 |
| T299 | 0.00 | 0.086 | 8.50 |  | 0.00 | 0.055 | 7.17 | T238 | 100.00 | 0.352 | 7.00 |  | 83.33 | 0.077 | 7.00 |
| T303 | 12.50 | 0.055 | 8.50 |  | 0.00 | 0.109 | 8.00 | T253 | 100.00 |  | 5.75 |  | 100.00 | 0.102 | 7.00 |
| T304 | 25.00 | 0.088 | 6.75 |  | 33.33 | 0.060 | 4.67 | T257 | 100.00 | 0.219 | 7.25 |  | 100.00 | 0.320 | 7.00 |
| T331 | 33.33 | 0.165 | 7.25 |  | 58.33 | 0.031 | 7.00 | T258 | 66.67 | 0.402 | 4.00 |  | 100.00 | 0.260 | 6.67 |
| T338 | 0.00 | 0.048 | 7.50 |  | 0.00 | 0.033 | 7.67 | T261 | 100.00 | 0.229 | 9.00 |  | 100.00 | 0.103 | 6.67 |
| T354 | 8.33 | 0.100 | 7.25 |  | 0.00 | 0.016 | 8.00 | T270 | 100.00 | 0.436 | 7.00 |  | 91.67 | 0.220 | 6.00 |
| T371 | 8.33 | 0.119 | 8.00 |  | 16.67 | 0.051 | 8.00 | T274 | 100.00 | 0.212 | 6.00 |  | 77.78 | 0.101 | 6.00 |
| T377 | 58.33 | 0.078 | 8.00 |  | 25.00 | 0.035 | 8.00 | T302 | 100.00 | 0.478 | 6.25 |  | 86.11 | 0.184 | 6.67 |
| T384 | 25.00 | 0.015 | 7.00 |  | 37.50 | 0.072 | 6.50 | T321 | 100.00 | 0.498 | 7.00 |  | 100.00 | 0.178 | 6.00 |
| T396 | 0.00 | 0.087 | 8.00 |  | 8.33 | 0.065 | 7.83 | T328 | 100.00 | 0.212 | 7.25 |  | 100.00 | 0.218 | 6.67 |
| T404 | 0.00 | 0.151 | 8.00 |  | 0.00 | 0.018 | 8.00 | T337 | 100.00 | 0.354 | 6.75 |  | 83.33 | 0.102 | 6.67 |
| T405 | 0.00 | 0.154 | 8.25 |  | 50.00 | 0.074 | 7.00 | T353 | 100.00 | 0.159 | 6.75 |  | 100.00 | 0.235 | 7.33 |
| T414 | 8.33 | 0.172 | 4.75 |  | 25.00 | 0.109 | 7.00 | T359 | 100.00 | 0.220 | 6.00 |  | 83.33 | 0.187 | 6.00 |
