# Supplementary material for: Localization of potato browning resistance genes based on BSA-seq technology
Source: PeerJ. 2024 Aug 6;12:e17831. doi: 10.7717/peerj.17831 (PMC11313402; doi:10.7717/peerj.17831)
Supplement: Table S4 [file peerj-12-17831-s006.docx]

Table S4 Function and annotation of candidate genes

|  | gene_ID | start | end | nr_annotation |
| --- | --- | --- | --- | --- |
| 1 | Soltu.DM.08G009080.1 | 24148809 | 24153252 | PREDICTED: uncharacterized protein LOC107060450 [Solanum tuberosum] |
|  | Soltu.DM.08G009080.2 | 24148809 | 24153153 | PREDICTED: uncharacterized protein LOC107060450 [Solanum tuberosum] |
| 2 | Soltu.DM.08G009100.1 | 24353907 | 24365325 | PREDICTED: probable phosphoinositide phosphatase SAC9 [Solanum tuberosum] |
| 3 | Soltu.DM.08G009110.1 | 24382527 | 24397536 | PREDICTED: probable phosphoinositide phosphatase SAC9 [Solanum tuberosum] |
| 4 | Soltu.DM.08G009160.1 | 24513703 | 24515707 | PREDICTED: putative F-box protein At5g55150 [Solanum pennellii] |
| 5 | Soltu.DM.08G009180.1 | 24553914 | 24555312 | PREDICTED: uncharacterized protein LOC102596535 [Solanum tuberosum] |
| 6 | Soltu.DM.08G009190.1 | 24559067 | 24560647 | hypothetical protein T459_07555 [Capsicum annuum] |
| 7 | Soltu.DM.08G009200.1 | 24623005 | 24623439 |  |
| 8 | Soltu.DM.08G009210.1 | 24753728 | 24763107 | PREDICTED: methylthioribose-1-phosphate isomerase [Solanum tuberosum] |
| 9 | Soltu.DM.08G009230.1 | 25060164 | 25064157 | PREDICTED: pentatricopeptide repeat-containing protein At5g42310, mitochondrial isoform X1 [Solanum tuberosum] |
|  | Soltu.DM.08G009230.2 | 25060126 | 25063995 | PREDICTED: pentatricopeptide repeat-containing protein At5g42310, mitochondrial isoform X1 [Solanum tuberosum] |
|  | Soltu.DM.08G009230.3 | 25060126 | 25064161 | PREDICTED: pentatricopeptide repeat-containing protein At5g42310, mitochondrial isoform X1 [Solanum tuberosum] |
| 10 | Soltu.DM.08G009280.1 | 25807041 | 25809000 | PREDICTED: chlorophyll(ide) b reductase NOL, chloroplastic [Solanum tuberosum] |
| 11 | Soltu.DM.08G009290.1 | 25817451 | 25836334 | chlorophyll(ide) b reductase NOL, chloroplastic isoform X2 [Solanum lycopersicum] |
| 12 | Soltu.DM.08G009310.1 | 25862144 | 25864160 | PREDICTED: probable 3-hydroxyisobutyryl-CoA hydrolase 3 [Solanum tuberosum] |
| 13 | Soltu.DM.08G009320.1 | 25878588 | 25883250 | PREDICTED: probable 3-hydroxyisobutyryl-CoA hydrolase 3 [Solanum tuberosum] |
| 14 | Soltu.DM.08G009350.1 | 26096933 | 26103061 | hypothetical protein PRUPE_3G016800 [Prunus persica] |
| 15 | Soltu.DM.08G009370.1 | 26445239 | 26448721 | PREDICTED: pentatricopeptide repeat-containing protein At1g08070, chloroplastic-like [Solanum tuberosum] |
| 16 | Soltu.DM.08G009390.1 | 26554158 | 26557390 | PREDICTED: uncharacterized protein LOC102580040 [Solanum tuberosum] |
| 17 | Soltu.DM.08G009400.1 | 26558710 | 26561538 | PREDICTED: WAT1-related protein At1g43650-like isoform X1 [Nicotiana tabacum] |
| 18 | Soltu.DM.08G009410.1 | 26563157 | 26563699 |  |
| 19 | Soltu.DM.08G009420.1 | 26604407 | 26608896 | PREDICTED: pentatricopeptide repeat-containing protein At3g53170 [Solanum tuberosum] |
|  | Soltu.DM.08G009420.2 | 26604407 | 26608896 | PREDICTED: pentatricopeptide repeat-containing protein At3g53170 [Solanum tuberosum] |
|  | Soltu.DM.08G009420.3 | 26604407 | 26608896 | PREDICTED: pentatricopeptide repeat-containing protein At3g53170 [Solanum tuberosum] |
|  | Soltu.DM.08G009420.4 | 26604407 | 26608896 | PREDICTED: pentatricopeptide repeat-containing protein At3g53170 [Solanum tuberosum] |
|  | Soltu.DM.08G009420.5 | 26604407 | 26608896 | PREDICTED: pentatricopeptide repeat-containing protein At3g53170 [Solanum tuberosum] |
|  | Soltu.DM.08G009420.6 | 26604407 | 26608896 | PREDICTED: pentatricopeptide repeat-containing protein At3g53170 [Solanum tuberosum] |
| 20 | Soltu.DM.08G009430.1 | 26663840 | 26664515 |  |
| 21 | Soltu.DM.08G009450.1 | 26720893 | 26729103 | ADP-ribosylation factor GTPase-activating protein AGD2-like isoform X1 [Solanum lycopersicum] |
